# The EndoPredict Gene-Expression Assay in Clinical Practice - Performance and Impact on Clinical Decisions

**DOI:** 10.1371/journal.pone.0068252

**Published:** 2013-06-27

**Authors:** Berit Maria Müller, Elke Keil, Annika Lehmann, Klaus-Jürgen Winzer, Christiane Richter-Ehrenstein, Judith Prinzler, Nikola Bangemann, Angela Reles, Sylvia Stadie, Winfried Schoenegg, Jan Eucker, Marcus Schmidt, Frank Lippek, Korinna Jöhrens, Stefan Pahl, Bruno Valentin Sinn, Jan Budczies, Manfred Dietel, Carsten Denkert

**Affiliations:** 1 Institute of Pathology, Charité University Hospital, Campus Mitte, Berlin, Germany; 2 Breast Center, Park Clinic Weissensee, Berlin, Germany; 3 Breast Center, Charité University Hospital, Campus Mitte, Berlin, Germany; 4 Gynecological Clinic, Berlin, Germany; 5 Gynecological Clinic, Berlin, Germany; 6 Department of Internal Medicine, Charité University Hospital, Campus Mitte, Berlin, Germany; 7 Breast Center, Johannes Gutenberg-University Mainz, Mainz, Germany; 8 Institute of Pathology, Neuruppin, Germany; The Chinese University of Hong Kong, Hong Kong

## Abstract

The validated EndoPredict assay is a novel tool to predict the risk of metastases of patients with estrogen receptor positive, HER2 negative breast cancer treated with endocrine therapy alone. It has been designed to integrate genomic and clinical information and includes clinico-pathological factors such as tumor size and nodal status. The test is feasible in a decentral setting in molecular pathology laboratories. In this project, we investigated the performance of this test in clinical practice, and performed a retrospective evaluation of its impact on treatment decisions in breast cancer. During one year, EndoPredict assays from 167 patients could be successfully performed. For retrospective evaluation of treatment decisions, a questionnaire was sent to the clinical partner. Regarding the molecular EP class, samples from 56 patients (33.5%) had a low-risk, whereas 111 patients (66.5%) showed a high-risk gene profile. After integration of the clinicopathological factors the combined clinical and molecular score (EPclin) resulted in a low-risk group of 77 patients (46.4%), while 89 (53.6%) had a high risk EPclin score. The EPclin-based estimated median 10-year-risk for metastases with endocrine therapy alone was 11% for the whole cohort. The median handling time averaged three days (range: 0 to 11 days), 59.3% of the tests could be performed in three or less than three days. Comparison of pre- and post-test therapy decisions showed a change of therapy in 37.7% of patients. 16 patients (12.3%) had a change to an additional chemotherapy while 25.4% of patients (n = 33) changed to an endocrine therapy alone. In 73 patients (56.2%) no change of therapy resulted. In 6.1% of patients (n = 8), the patients did not agree to the recommendation of the tumor board. Our results show that the EndoPredict assay could be routinely performed in decentral molecular pathology laboratories and the results markedly change treatment decisions.

## Introduction

Breast cancer is still the most prevalent cancer type in women, accounting for 29% of all cancer cases in women in 2013. In addition it is the second leading cause of cancer related death in women [1]. Adjuvant treatment decisions are based on various national and international guidelines and tools [2], [3], [4].

In addition to the known relevant clinicopathological prognostic factors like e.g. large tumor size (>2 cm), lymph node metastasis or HER2 gene amplification [5], [6], genomic multigene assays can be used as additional tools to assist treatment decisions and to avoid under- or overtreatment by estimation of the biological tumor behavior [7], [8]. These multigene signatures were discussed on the 13^th^ St Gallen International Breast Cancer Conference 2013 [9]. In this context, especially the identification of patients with ER-positive HER2-negative breast cancer with intermediate or high risk of recurrence defined by conventional clinicopathological features but low risk defined by multigene assays seems to be important for therapy decisions regarding chemotherapy and endocrine therapy. Furthermore, selected prognostic gene expression arrays that can be used in daily practice are listed in the WHO 2012 blue book [10].

The most widely used assays in this regard are OncotypeDX® (Genomic Health, Inc., Redwood City, CA, USA) and Mammaprint® (Agendia BV, Amsterdam, The Netherlands) [11], [12].

The validated EndoPredict assay (EP) is a novel tool to predict the risk of metastases of patients with estrogen receptor positive (ER positive), HER2 negative breast cancer treated with endocrine therapy alone. Both risk scores were validated in two large independent clinical trials (ABCSG-6: n = 378, ABCSG-8: n = 1324) [13]. This assay can be performed on formalin-fixed paraffin-embedded tissue [13]. It provides additional prognostic information to standard pathological factors including ki67 and improves risk classification from common clinical guidelines [14]. In a recent review [15], the EndoPredict assay has been assigned the level of evidence 1 according to Simon et al. [16], this level of evidence is identical e.g. to the Oncotype DX® recurrence score.

The EndoPredict assay has been designed to integrate genomic and clinical information and therefore includes clinico-pathological factors such as tumor size and nodal status. From the view of diagnostic molecular pathology, the EndoPredict assay is an example of a new generation of molecular assays, since it is the first multigene expression assay which is very suitable for decentralized testing in specialized molecular pathological laboratories as shown by a round-robin trial [17]. Analytical performance characteristics and the robustness of the test in a molecular-pathological laboratory has been published [18].

The test has been introduced in Germany as a new diagnostic tool in August 2011. The Charité University Hospital has been the first diagnostic molecular pathology laboratory that established the EndoPredict assay in routine diagnostic and has performed a large series of EP assays during the first year.

In this project, we investigated the performance of this test in clinical practice, and performed a retrospective evaluation of the impact of this new test on treatment decisions in breast cancer.

## Methods

### Study Population and Clinicopathological Parameters

Within one year (August 2011– July 2012), we received a total of 168 diagnostic requests to conduct the EndoPredict assay at the Institute of Pathology at the Charité University Hospital in Berlin, Germany. EndoPredict assays from 167 patients could be successfully performed, for one single sample the RNA extraction was not possible. The formalin-fixed paraffin-embedded (FFPE) tissue samples derived from female patients with primary invasive estrogen receptor (ER) positive, HER2 negative breast cancer. The median age at time of diagnosis was 54 years (range: 30–78 years), the median age in the subgroup with therapy data was 55 years (range: 30–76 years). The clinicopathological data (tumor size, pT, nodal status, grading, Ki67) were extracted from the pathological reports. Concerning Ki67, the cutoff point was extracted from the St. Gallen guidelines [2]. Table 1 gives an overview on these factors of the patients. For retrospective evaluation of treatment decisions, a questionnaire was sent to the clinical partner. The questionnaire consisted of two questions each with two possible answers regarding the treatment decisions (endocrine therapy alone vs. endocrine therapy together with chemotherapy) before and after the EndoPredict – test.

**Table 1:** Patients characteristics.

Characteristic	number of all patients	%	subgroup with therapy data	%
**All**	167	100	130	100
**Tumor size (mm)**				
pT1a	1	0.6	1	0.8
pT1b	17	10.2	15	11.5
pT1c	68	40.7	51	39.2
pT2	67	40.1	53	40.8
pT3	14	8.4	10	7.7
**Nodal status** *				
pN0	103	62.1	81	62.3
pN1	59	35.5	47	36.1
pN2a	2	1.2	1	0.8
pN3a	2	1.2	1	0.8
**Histological grade** *				
G1	18	11.3	15	12.2
G2	113	71.1	89	72.4
G3	28	17.6	19	15.4
**Hospital**				
intern	65	38.9	46	35.4
extern	102	61.1	84	64.6
**Tumor proliferation** ^∗^				
Ki67<14%	67	54.9	50	52.6
Ki671≥14%	55	45.1	45	47.4

* = Not all of the data were available for all patients.

### Ethical Statement

For this study, only existing data from routine diagnostic procedures were used, which were performed with informed consent as part of routine patient care. No additional tissue-based evaluations were performed. Therefore, no ethics committee approval was needed, based on the legal requirements in Berlin (Landeskrankenhausgesetz § 25.1, version 18.09.2011) which allow evaluation of existing diagnostic data.

### RNA Extraction and Assessment of the EndoPredict Score

The invasive cancer was verified on a hematoxilin-eosin-stained slide before RNA was extracted, in case of a tumor area <30%, a macrodissection was done before RNA extraction. Usually, one 5 µm slide was used. In case of a lower tumor content, more than one 5 µm slide was used. The extraction of total RNA was carried out using a fully automatic method as described previously [19], [20], [21], alternatively manually according to the same protocol. The EndoPredict assay analyzes the expression levels of eight genes of interest (BIRC5, UBE2C, DHCR7, RBBP8, IL6ST, AZGP1, MGP and STC2) as well as three reference genes [13]. PCR was performed as described before [17]. Relative expression levels of each gene of interest as well as EP and EPclin scores were calculated as described previously 13 using a web-based implementation (http://forschung.medizin.uni-mainz.de/epreport/) to process analytical PCR results into test results. Referring to this, the EPclin score combines the EP score with tumor size and nodal status resulting in a molecular-clinicopathological hybrid score. Finally, samples were classified as low or high risk of distant metastasis according to the predefined cutoff value of 5 (molecular risk score EP) respectively 3.3 (integrated molecular and clinical risk score EPclin) [13].

### Statistical Methods

The statistical analysis was done using SPSS Statistics Version 18 (IBM, Armonk, USA). The correlation between EP score and tumor grade was analyzed using the Jonckheere-Terpstra test for trends. The correlation between EP and proliferation activity was analyzed using the Wilcoxon-Mann-Whitney test. The graphics were generated with GraphPad Prism 5 (GraphPad Software, Inc., La Jolla, CA, USA).

## Results

### Test Performance and Distribution of Risk Groups

During one year, we routinely performed EndoPredict tests from a total of 167 patients in our molecular pathology laboratory.

**Figure 1:**
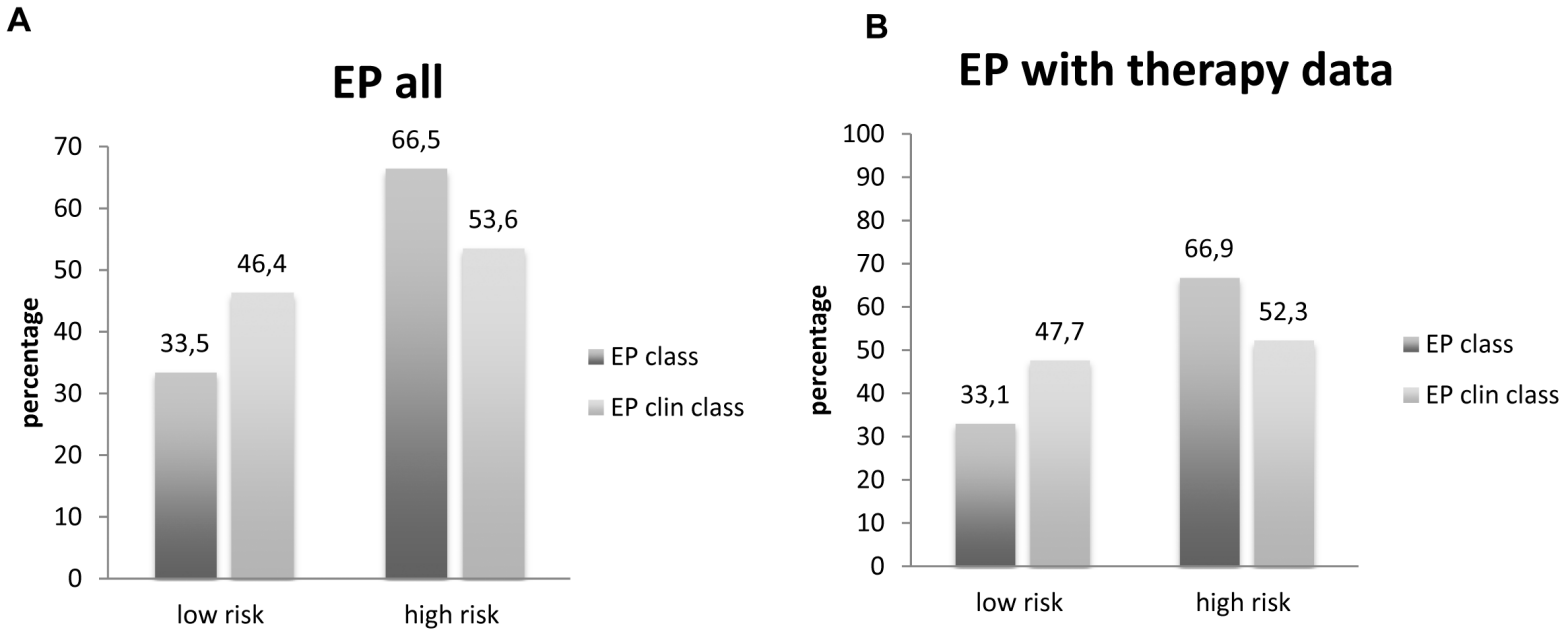
Distribution of EP class and EP clin class. Distribution of EP class and EP clin class of all included EndoPredict assays (A). Distribution of EP class and EP clin class of a subgroup of patient for which therapy decision data were available (B).

Regarding the molecular EP class, samples from 56 patients (33.5%) had a low-risk, whereas 111 patients (66.5%) showed a high-risk gene profile (Fig. 1A). After integration of the clinicopathological factors (tumor size, nodal status) the combined clinical and molecular score (EPclin) resulted in a low-risk group of 77 patients (46.4%), while 89 (53.6%) had a high-risk EPclin score. For one patient, the combined clinical and molecular score (EPclin) could not be analyzed due to the unknown nodal status. The EPclin-based estimated median 10-year-risk for metastases with endocrine therapy alone was 11% for the whole cohort. The estimated median risk for the EPclin low group was 7%, for the EPclin high risk group it was 19%.

**Figure 2:**
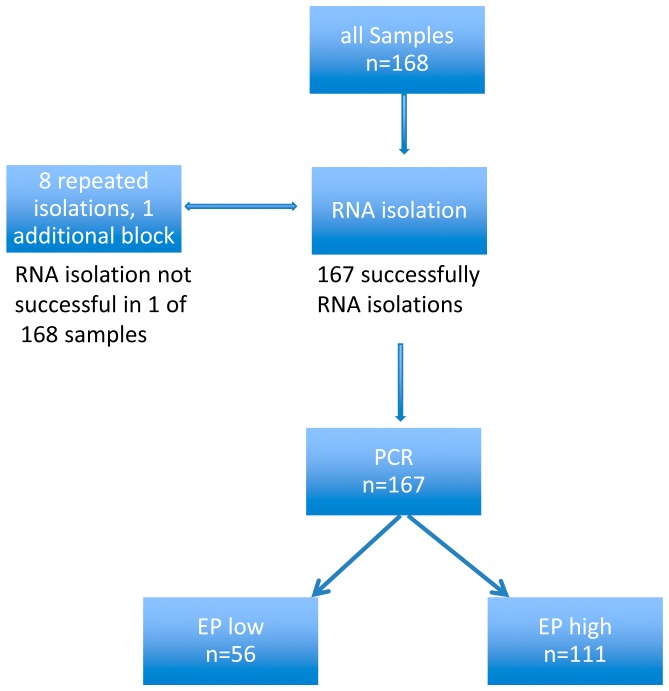
Test performance of the EndoPredict assay during one year. 167 samples could successfully analysed during one year.

The median handling time averaged three days (range: 0 to 11 days). 59.3% of the test could be performed in three or less than three days. The reasons for delay included missing clinical information as well as the need for repeated RNA extraction (8 cases). For one case, an additional paraffin block had to be requested from the local pathologist (Fig. 2).

### Comparison of EndoPredict with Standard Clinical Parameters

**Figure 3:**
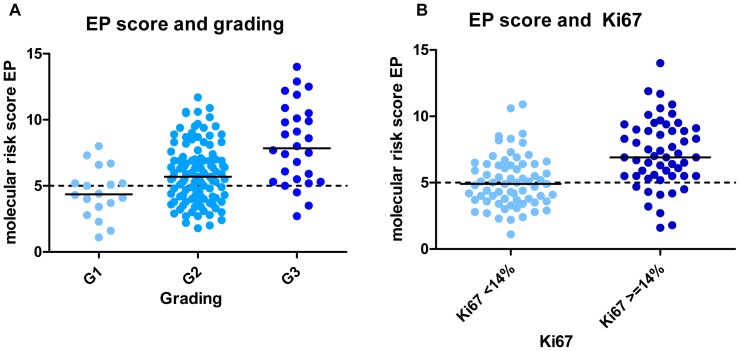
Distribution of the molecular risk score EP related to the histological grade and mitotic index. Distribution of the molecular risk score EP related to the histological grade (A) as well as to the mitotic index (B). The continuous line revealed the median, the dotted line highlighted the cutoff point of the molecular risk score EP. The cutoff point of ki67 was extracted from the St. Gallen guidelines [2].

The median of the EP score increased from 4.5 to 5.9 and 8.1 for tumors with histopathological grade G1, G2 and G3 (p = 7.6E-06). Further, the EP score had a higher median of 6.9 in tumors with high Ki67-index compared to 4.9 in slowly proliferating tumors (p = 1.5E-06). Figure 3 gives an overview of the distribution of the molecular risk score EP depending on the histological grade as well on the Ki67-index.

### Evaluation of the Impact of EndoPredict on Changes in Therapy Decisions

The information on treatment decisions was retrospectively collected using a standardized questionnaire. This therapy information was available from 130 (77.8%) of the 167 patients. Of those patients, 62 (47.7%) had a low risk combined clinical and molecular score (EPclin), the remaining 68 patients (52.3%) were EPclin high risk (Fig. 1B).

Before the EndoPredict assay, 47 patients (36.2%) had been scheduled for endocrine therapy alone. In contrary, for 83 patients (63.8%) a combination of endocrine therapy and chemotherapy had been planned. After the results of the EndoPredict assay were available, the number of patients with endocrine therapy alone was increased to 67 (51.5%) and only 62 patients (47.7%) were scheduled for a combination therapy of chemotherapy and endocrine therapy. For one patient the therapy decision after the EndoPredict assay was unknown because of the prior desire of the patient for another therapy.

**Figure 4:**
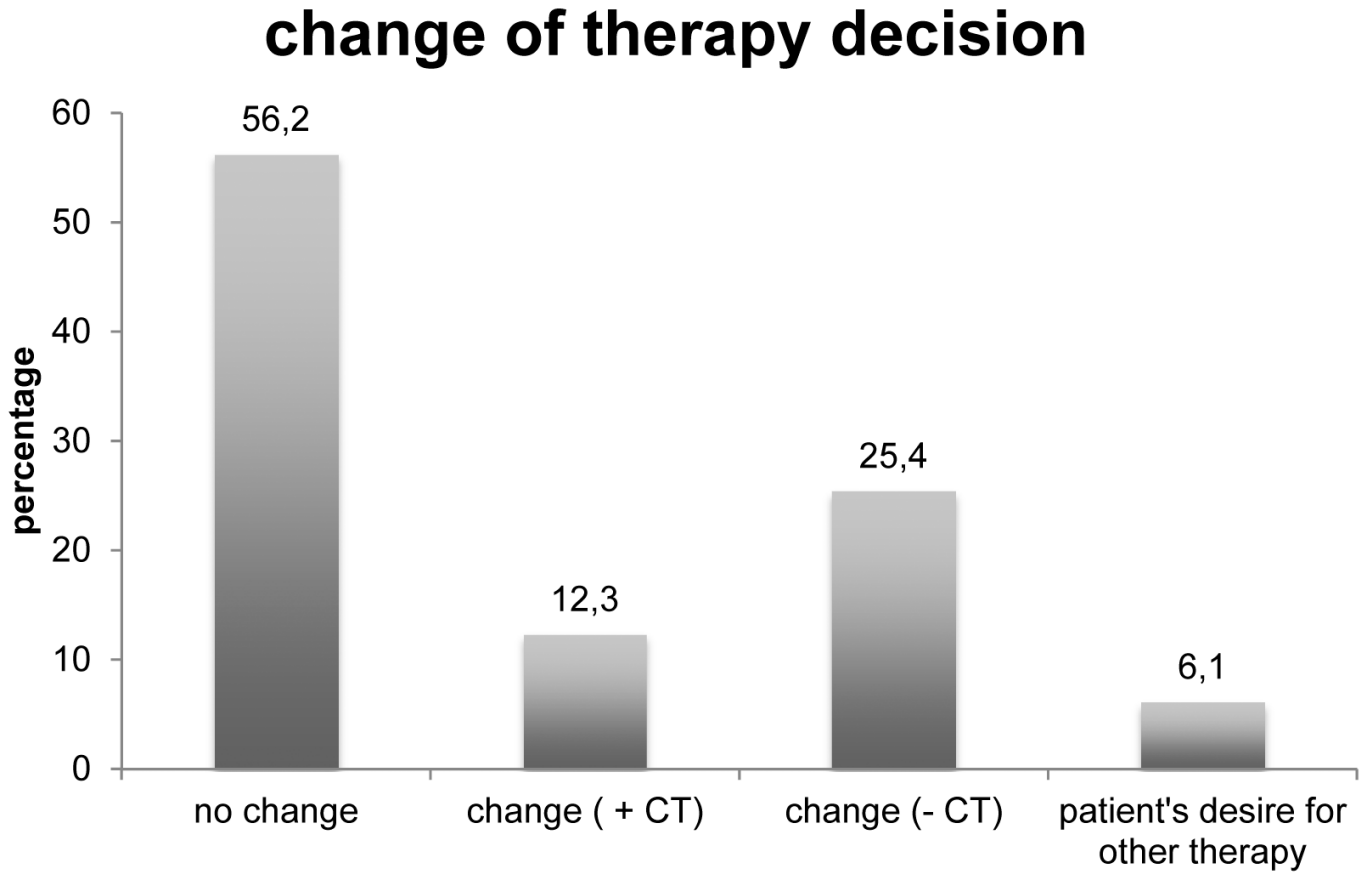
Changes in therapy decisions. Changes in therapy decisions regarding the decision before and after the EndoPredict assay.

**Figure 5:**
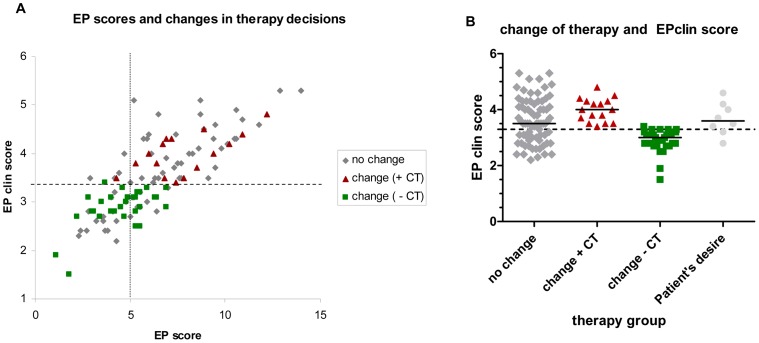
Therapy decision related to the molecular risk score EP and the combined clinical and molecular score (EPclin). Association between the molecular risk score EP, the combined clinical and molecular score (EPclin) and therapy decision (A). The group of patient’s desire for other therapy was excluded. The dotted vertical line marks the cutoff values of the molecular risk score EP, the broken horizontal line marks the cutoff value of the combined clinical and molecular score (EPclin). Additionally, the therapy decisions related to the combined clinical and molecular score (EPclin) are shown (B). The broken horizontal line marks the cutoff value of the combined clinical and molecular score (EPclin), the continuous line indicates the median.

Comparison of pre- and post-test therapy decisions showed a change of therapy in 37.7% of patients. In detail, for 16 patients (12.3%) it was decided to administer an additional chemotherapy based on the results of the EndoPredict assay. On the other hand, the therapy of 25.4% of patients (n = 33) was reduced to endocrine therapy alone. In 73 patients (56.2%) no change of therapy resulted from the EndoPredict result. Additionally, 8 patients did not agree to the recommendation of the tumor board after the EndoPredict assay. Figure 4 gives an overview about all changes in therapy decisions. Figure 5 depicts the changes of therapy decisions depending on the molecular risk score EP and the combined clinical and molecular score (EPclin).

## Discussion

Our study demonstrates that the EndoPredict assay can be reliably performed in a routine molecular pathology laboratory in daily practice. The test could be successfully performed in 99% of all samples. More than 50% of tests were performed in three or less days.

This is the first analysis of changes in therapy decisions based on the EndoPredict assay. In over one third (37.7%) the results of the EndoPredict assay lead to a change of planned therapy. For a quarter of patients (25.4%) the originally planned chemotherapy could be omitted based on the result of the multi-gene assay.

Our results are in line with those observed for the prognostic Oncotype® DX 21-gene assay [22], [23]. Similar to the current study, they observed a change of treatment recommendations in about thirty percent (31.5% resp. 32%). Comparable with our results, most of the changes were caused by reduction from chemotherapy plus endocrine to endocrine therapy alone (22.5% resp. 21%). A change from endocrine to chemo-endocrine therapy was observed in 3.4% [22] to 11% [23]. Therefore, despite the limitation of our study that therapy changes were only retrospectively assessed, the results are comparable with other reports. Recently, it was shown by Blohmer et al. according to other published studies that the treatment decision using the OncotypeDX® on adjuvant therapy leads to a reduction of costs as compared to costs without this molecular test [24]. The EndoPredict assay can be successfully done in decentral molecular pathology laboratories [17] with a median handling time of three days.

Furthermore, the results of the OncotypeDX® are divided in three groups: low Recurrence Score (RS), intermediate RS and high RS, whereas the EndoPredict results in only two groups (low risk, high risk). In contrast to most of the other multigene assays, the EndoPredict assay includes the relevant clinicopathological factors tumor size and nodal status which are known to be essential for assessing the biological behavior of breast cancer. The interpretation of the test results was in most cases straightforward, test results near the cutoff point were intensively discussed with the clinicians and the patients. From the point of view of a clinician having to decide whether to recommend additional adjuvant chemotherapy to a patient or not, this dichotomization of risk is helpful.

Recently, it was shown by Blohmer et al. according to other published studies that the treatment decision using the OncotypeDX® on adjuvant therapy leads to a reduction of as compared to costs without this molecular test [24].

One limitation of our study is the retrospective assessment of the therapy changes. In addition, the EndoPredict test was a completely new test and the clinicians did not have any experience with the test at the time our analysis started. Therefore in the beginning the clinicians were not prepared to change therapy based on the test, which might underestimate the changes in therapy once the test is fully integrated in the diagnostic workup.

As a conclusion, our results show that the EndoPredict assay could be routinely performed in a decentral molecular pathology laboratory. The results of this multi- gene assay markedly change treatment decisions supporting clinical utility of this new diagnostic method. Based on the comprehensive clinical and analytical validation data and our results from clinical routine diagnostics, we suggest that implementation of this test could be very helpful as an additional tool for treatment decisions in breast cancer patients in clinical practice.
